# Multi-scale modelling of mouse tibia loading reveals the critical role of muscle insertion surfaces

**DOI:** 10.1007/s10237-026-02078-2

**Published:** 2026-06-03

**Authors:** Thomas Valerio, Saira Mary Farage-O’Reilly, James P. Charles, Enrico Dall’Ara

**Affiliations:** 1 Division of Clinical Medicine, School of Medicine and Population Health, University of Sheffield;, Sheffield, UK; 2 Insigneo Institute for in silico Medicine, University of Sheffield, Pam Liversidge Building; Mappin St, S1 3JD (UK), Sheffield, UK; 3 Institute of Life Course & Medical Sciences, University of Liverpool, Liverpool, UK

**Keywords:** Micro-finite element analysis, Strain energy density, Musculoskeletal modelling, Muscle insertion surface, Bone mechanoregulation

## Abstract

The definition of the muscle insertion surface (MIS) is often simplified in micro-finite element (micro-FE) models despite its potential impact on the predictions of mechanical properties that are relevant to bone adaptation. This study investigated the effect of MIS size on local strain energy density (SED) in the mouse tibia. Micro-FE models of six mouse tibiae were developed from micro-CT images, and loading conditions were derived from musculoskeletal simulations of trotting gait. For the seven muscles with the highest contributions to tibial loading, three MIS configurations were tested: full anatomical surface, 50% reduced surface, and a single-node application. A total of 198 micro-FE models were run, both for individual muscle applications and multi-muscle configurations at key instants of the stance phase. SED values were compared across MIS conditions using the mean absolute difference, normalised by the median, mean, or 95th percentile of the SED. Significant differences in SED were observed between MIS configurations, with the largest effects near the MIS and boundary conditions. The effect of MIS modification on SED values was heterogeneous across the different muscles, highlighting the importance of anatomical features. Even with multiple muscle loading, SED differences exceeded 20% in most comparisons. These results demonstrate that simplifications of the MIS, especially to single-node applications, can considerably alter local SED predictions and are likely to compromise the accuracy of bone adaptation models. Accurate representation of the MIS is therefore essential for reliable mechanoregulation analysis and should be prioritised in multiscale models of skeletal loading.

## Introduction

Osteoporosis is a progressive skeletal disorder characterised by decreased bone mass and microarchitectural deterioration, leading to reduced bone strength and increased fracture risk (Gkastaris et al. 2020; Lorentzon et al. 2022; Rachner et al. 2011). It poses a major public health challenge, especially in ageing populations, due to its high prevalence (Salari et al. 2021) and the significant burden associated with osteoporotic fractures (Lorentzon et al. 2022; Shen et al. 2022). Despite the availability of anti-osteoporosis (anti-resorptive or bone anabolic) pharmacological treatments, their long-term efficacy and safety remain limited, often failing to fully restore bone integrity (Shane et al. 2014; Yeam et al. 2018). One reason for this limitation lies in the complex mechanisms underlying bone remodelling, which are influenced by different mechanical and biological stimuli (Bonewald 2011; Frost 2003; Martin et al. 1998).

Mechanical loading plays a central role in bone adaptation, as it regulates the bone remodelling process through local mechanical stimuli such as effective strain, principal strain, strain energy density (SED) (Cheong et al. 2021), and gradients of SED (Scheuren et al. 2020). To better understand these processes, integrated experimental and computational approaches have been developed that combine motion capture, multi-body dynamic modelling (MBD), and finite element (FE) modelling to estimate in vivo mechanical loading on human bones (Altai et al. 2021; Phillips et al. 2015; Valente et al. 2017). However, the development of such approaches to the mouse is limited due to experimental and computational challenges, despite the large amount of preclinical data available for assessing new combined treatments.

Mouse models are widely used in bone research to study new interventions for skeletal diseases, because of their genetic manipulability, relevance to human bone physiology and (re)modelling, and the possibility of controlling their environment (Elefteriou and Yang, 2011). While in mice, the bone microstructure can be well characterised by using in vivo micro-computed tomography (micro-CT) (Bouxsein et al. 2010; Van’T Hof and Dall’Ara, 2025), other parameters, such as the joint forces, cannot be measured experimentally. High-resolution micro-CT images can be used to create high-fidelity personalised biomechanical models of individual bones (micro-finite element, micro-FE, models). However, developing accurate multiscale biomechanical simulations in mice remains challenging due to their small size, limited experimental data, and high computational costs. For example, only a few studies have investigated muscle forces in mice during trotting (Akay et al. 2014; Charles et al. 2018, 2024). While these studies have highlighted the variability and importance of the different muscle forces in the mouse hindlimb, their effect on the local bone deformation, the main trigger for bone adaptation, remains unexplored.

Micro-FE models have been developed over the past decade to estimate the bone mechanical properties and local deformation of the mouse tibia. These models have been shown to accurately predict the apparent mechanical properties and local deformation of bone (Oliviero et al. 2018; Oliviero et al. 2021a, b), and are highly sensitive to the direction of joint forces (Farage-O’Reilly et al. 2024). However, their use remains predominantly limited to simulating simple knee joint forces, ignoring the effect of muscle forces, which may affect the bone deformation and consequent bone adaptation to mechanical stimuli. Merging MBD and micro-FE models for the mouse hindlimb is essential to better understand how bone adaptation is affected by realistic mechanical loading driven by muscle and joint forces. Nevertheless, a critical aspect of such implementations is accurately representing how muscle forces are transmitted to the bone, especially the area over which these forces act, known as the muscle insertion surface (MIS). While muscle forces in FE models are often applied to a single node or small area (Seo et al. 2014; Speirs et al. 2007), this simplification may significantly affect the local mechanical stimuli (i.e. SED) predicted within the bone, as was previously demonstrated for the human femur (Polgar et al. 2003). However, the effect of this simplification has never been investigated for mouse models.

The aim of this study was to investigate the sensitivity of MIS on the local SED values estimated from micro-FE models of the mouse tibia. This study contributes to refining multiscale modelling pipelines for murine studies and offers guidance to improve the physiological relevance of biomechanical simulations in mice.

## Methods

### Overview of the approach

**Fig. 1:**
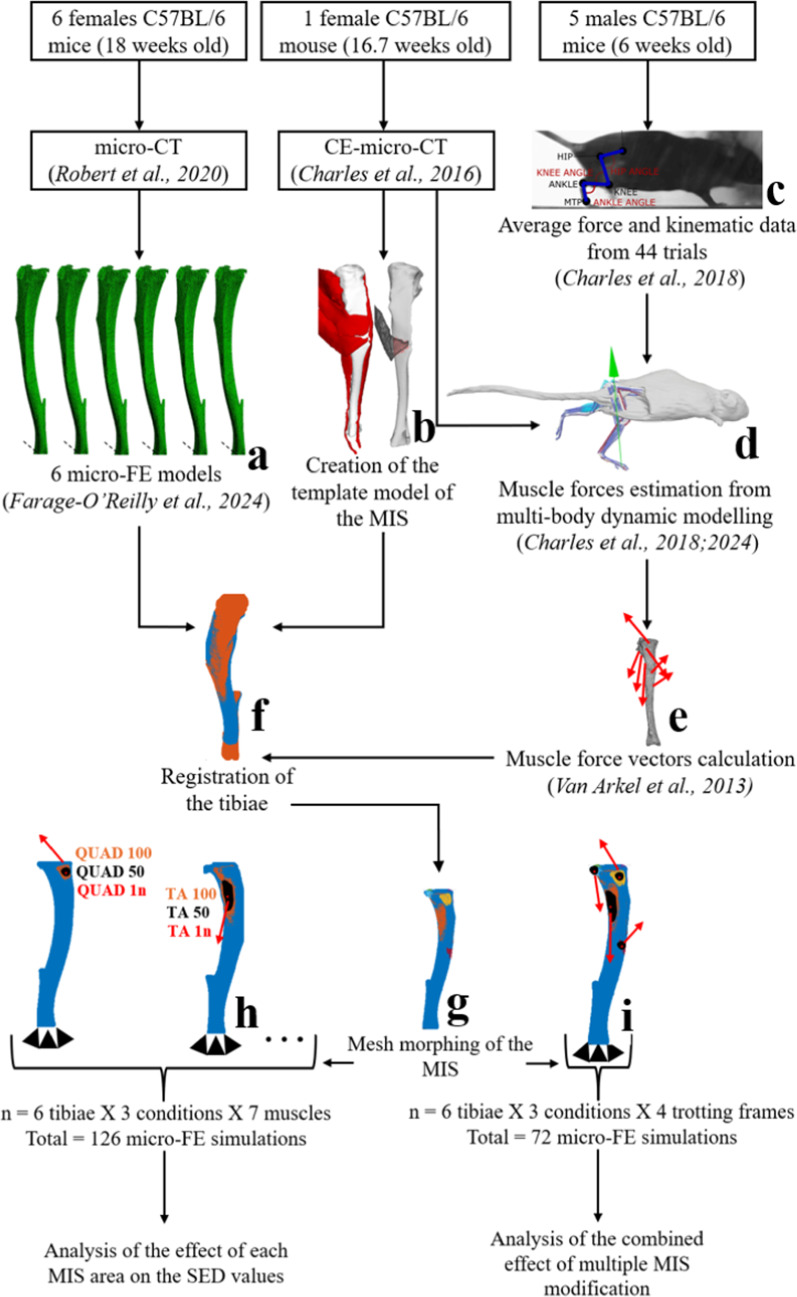
Summary of the modelling pipeline used in this study to evaluate the effect of the muscle insertion surface (MIS) on the strain energy density (SED) values calculated with the micro-FE models of the mouse tibia

The main steps of the approach are briefly illustrated in Fig. 1, with further details provided below. Subject-specific micro-FE models of the right tibia from six mice were created using previously collected micro-CT images. The MIS for each model was determined by registering a template based on contrast-enhanced micro-CT (CE-micro-CT) images that clearly showed muscles and their insertions. Each MIS was manually defined from these images, then a mesh morphing technique was applied to transfer the MIS onto each micro-FE model. Realistic muscle forces were estimated by combining MBD modelling with previously acquired experimental data (Charles et al. 2018, 2024) and applied to each micro-FE model. The effect of distributing muscle forces over three different MISs on the local SED values predicted by the micro-FE models was studied for each muscle. This sub-study (Fig. 1h) aimed at identifying for which muscles the definition of realistic or simplified MIS played an important role in the calculation of the SED values and distributions from the micro-FE model. Additional simulations incorporating all major muscle forces were then performed to evaluate the effect of combined muscle forces on the local SED values and how they were affected by modifying multiple MISs at the same time. This sub-study (Fig. 1i) aimed at understanding the potential interaction between different MISs for different muscles when they were inputted in more complex (and more realistic) micro-FE models including multiple loading forces.

### Calculation of muscle forces

A previously developed MBD model of the mouse hindlimbs (Charles et al. 2018, 2024) was used to estimate the muscle forces acting on the tibia during trotting. Both hindlimbs were included, and each limb consisted of 42 muscles and 7 degrees of freedom. The model was driven by using one set of joint angles of the mouse hindlimbs representing average trotting kinematics measured on 44 trials involving five C57BL/6 male six-week-old mice (Charles et al. 2018). Net joint moments around each unlocked degree of freedom were predicted using inverse dynamics in Opensim v4.5 (Seth et al. 2018). The muscle forces of the 23 muscles having an insertion point on the tibia were then predicted using static optimisation, with the objective function of minimising the square of the muscle activations sum at each time of the trotting phase. The applied external loads (i.e. ground reaction forces) for the static optimisation simulation were derived from (Charles et al. 2018).

**Fig. 2:**
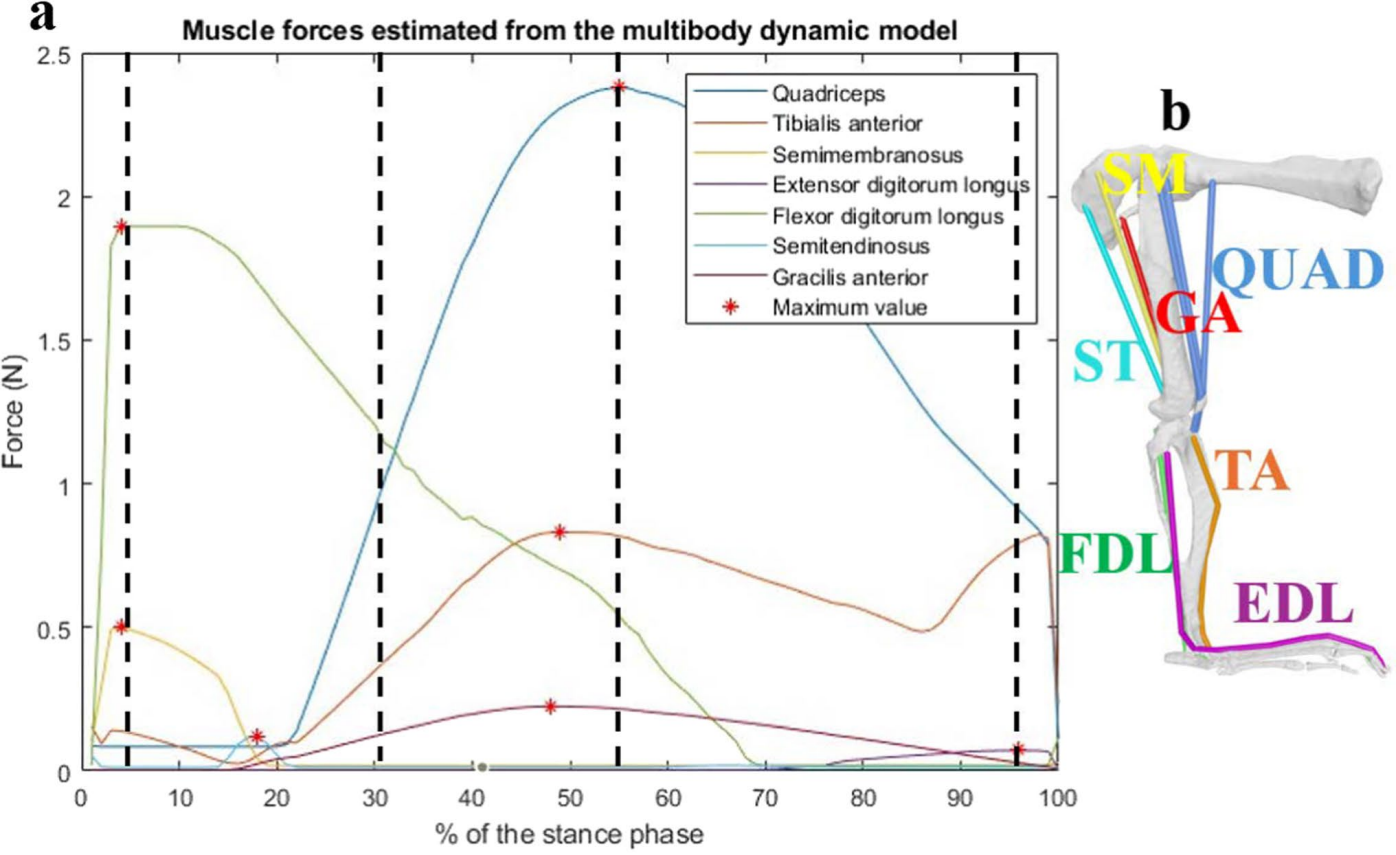
Evolution of the muscle forces during the stance phase of the mouse for the seven muscles selected in this study (**a**) and their respective anatomical location (**b**) in the model of (Charles et al. 2018). These muscle forces were estimated at each frame of the trotting phase of the mouse by using the MBD model presented by (Charles et al. 2016; 2018). The input data used for estimating these forces were a set of joint angles and ground reaction forces representative of the average trend observed in 44 trials. The stance phase corresponds to the period when the right foot of the mouse is in contact with the force plate placed on the ground to measure the ground reaction force. The maximum values visible on the graph (**a**) correspond to the force applied on each micro-FE model used in this study. The dashed lines represent the instants selected to test the effect of multiple MIS. The acronym used to indicate the location of the muscles (**b**) corresponds to the acronym previously defined

For the sensitivity analysis, only the seven muscles that individually contributed more than 5% to the total muscle force were considered: tibialis anterior (TA), semimembranosus (SM), flexor digitorum longus (FDL), extensor digitorum longus (EDL), semitendinosus (ST), gracilis anterior (GA), and the quadriceps (QUAD, considered as the combined group of rectus femoris, vastus medialis, vastus lateralis, and vastus intermedius). The forces produced by these seven muscles during the stance phase and the timing of their peak magnitudes are shown in Fig. 2. A sensitivity analysis of the MIS area was performed for each of these muscles to assess its influence on the SED distributions computed using the micro-FE models.

### Generation of micro-FE models of the mouse tibia

Micro-FE models were developed from in vivo micro-CT images acquired in a previous study (Roberts et al. 2020), validated against biomechanical experiments (Oliviero et al. 2018; Oliviero, Roberts, et al., 2020),Query and used to evaluate the effect of different joint loading conditions on the structural and local properties of the mouse tibia (Farage-O’Reilly et al. 2024, 2025). Briefly, the right tibiae of six female C57BL/6 mice were scanned at 18 weeks of age using a VivaCT80 scanner (Scanco Medical, Brütisellen, Switzerland), following ovariectomy performed at 14 weeks of age (see Roberts et al. 2020 for more information about scanning parameters). Bone voxels were segmented from the background using a single-level thresholding method, with the threshold set as the midpoint between the peaks corresponding to bone and background in the histogram of image attenuation values (Cheong et al. 2021; Oliviero et al. 2018). Micro-FE models were generated by converting each bone voxel into a linear 8-node hexahedral element. Voxels not connected to the main structure through at least one surface were removed. Bone was modelled as an isotropic, linear elastic, and homogeneous material with a Young’s modulus of 14.8  GPa and a Poisson’s ratio of 0.3. The SED distribution (nodal values) was calculated in each tibia in function of the loading obtained during the stance phase, by applying the muscle forces on the corresponding MIS (Sect. 4).

### Determination of the MIS area

The MIS area of the seven muscles selected in this study was determined from a 3D model of the bones and muscles of the mouse right hindlimb, obtained from CE-micro-CT scan (Charles et al. 2016). The CE-micro-CT image was acquired from a cadaveric C57BL/6 female 117 days old mouse (between 16 and 17 weeks of age, similar to the age of the mice used to create the micro-FE models in this study). The 3D model was previously obtained by manually segmenting the images and by creating a triangular surface mesh (average triangle edge = 0.1 mm) by using the Mimics software (Materialise Inc., Leuven, Belgium) (Charles et al. 2016). The triangular faces of the surface mesh of the tibia close to the muscle insertion visible in the CE-micro-CT image were manually selected (Fig. 1b). A template model of the mouse tibia with the corresponding MIS was thus created from this process. To determine the same MISs in the six micro-FE models of the mouse tibiae selected in this study, the template model was rigidly registered to each micro-FE model (Fig. 1). The registration was performed by using the iterative closest point algorithm (Besl & McKay 1992) implemented in MATLAB R2024a. Given the similarity in age (18 weeks vs. 16–17 weeks) and tibia length (average difference of 1.3%) between the mice used for the micro-FE models and those scanned with CE-micro-CT imaging, no scaling correction was applied. To compensate for any geometrical differences between the tibiae, the nodes of the micro-FE models closest to the corresponding MIS in the CE-micro-CT image were identified by setting a distance threshold between 0.1 mm and 0.3 mm. In order to analyse the effect of the MIS area on the micro-FE outputs, three different MISs were identified for each considered muscle: entire MIS (MIS_100%), a MIS shrunk by 50% (MIS_50%), or the node located at its centre (MIS_1n).

### Micro-FE model simulations

For each of the seven selected muscles, micro-FE simulations were performed using three different MIS conditions, on each of the six mouse tibiae. For each muscle, the maximum force estimated by the MBD model during the stance phase of trotting was applied (see Fig. 2), with all other muscle forces set to zero. The orientation of the applied muscle force was determined using an OpenSim plugin (Van Arkel et al. 2013), which calculates the orientation of the effective force applied to the bone, considering all muscle trajectories. A total of 126 micro-FE simulations were run (6 tibiae, 3 MISs, 7 muscles). Moreover, in order to evaluate the potential interactions between muscles, three simulations for each tibia for the different MISs were run by including all selected muscles at four different instants of the stance phase: at 5% of the stance phase (when the FDL muscle force is the highest), at 30% of the stance phase (when the QUAD, FDL, TA, and GA muscle forces are high), at 55% of the stance phase (when the QUAD muscle force is the highest), and at 95% of the stance phase (when the EDL muscle force is the highest) (Fig. 2). This analysis included an additional 72 simulations (6 tibiae, 3 MISs, 4 instants of the stance phase), leading to a total of 198 simulations for the whole study. All simulations were run using Abaqus 2018 (Dassault Systèmes Simulia, RI, USA) on the University of Sheffield; High Performance Computing Clusters (Stanage). From each simulation, the nodal SED values were calculated. Considering that the mesh of the three micro-FE models created for one mouse was the same for each of the three considered MISs, the mean absolute differences of the nodal SED values (MAD_SED) between two of the three different conditions were calculated (MIS_100% vs MIS_50%; MIS_50% vs MIS_1n; MIS_100% vs MIS_1n). To prevent artefacts due to the boundary conditions from influencing the analysis, the nodes corresponding to 150% of the original MIS were excluded from the calculation of SED values. To compare the results across mice and for different muscles, the MAD_SED values were normalised using three metrics: the median SED value calculated for all nodes in one reference condition; the mean SED value calculated for all of the nodes in one reference condition; the 95th percentile of the SED values calculated for all nodes in one reference condition. The last two normalisation metrics were chosen to evaluate the effect of different MIS values on the highest values of SED, more likely to be associated with bone apposition (Cheong et al. 2021). In all cases, the MIS with the largest number of nodes (MIS_100% or MIS_50%) was selected as the reference condition. The normalised difference was then averaged across all nodes of the model and compared for the different conditions. This process is summarised by the following equation, with N being the number of nodes, $\overline{{{\mathrm{SED}}_{1}^{i} }}$ being the SED value for the node *i* for the first condition, $\overline{{{\mathrm{SED}}_{2}^{i} }}$ being the SED value for the node *i* for the second condition, and $\overline{{{\mathrm{SEDref}}}}$ being normalisation factor (median, mean or 95th percentile of the SED) for the reference condition:

1 $$\Delta_{{{\mathrm{SED}}}}^{\% } = \frac{1}{N}\mathop \sum \limits_{i = 1}^{N} \left( {\frac{{\left| {\overline{{{\mathrm{SED}}_{1}^{i} }} - \overline{{{\mathrm{SED}}_{2}^{i} }} } \right|}}{{\overline{{{\mathrm{SEDref}}}} }}} \right) \times 100$$

This difference in SED values ${\Delta}_{\mathrm{S}\mathrm{E}\mathrm{D}}^{\%}$ was calculated for each muscle for which the effect of the single muscle MIS was evaluated, and for the simulations performed by including multiple muscles. These metrics were then averaged across the different mouse tibiae and means and standard deviations (SD) were calculated. For each tested configuration (each muscle for each tibia, and each instant for each tibia for the multiple simulations),

### Statistical Analysis

Friedman tests were performed to test if the configurations MIS_100%, MIS_50%, and MIS_1n had a significant impact on the SED values. The distribution obtained for each simulation was also tested to confirm if the distribution followed a Pareto distribution or not. This was done by calculating the Maximum Likelihood Estimation followed by the Kolmogorov–Smirnov test, as recommended by the Clauset-Shalizi-Newman procedure (Clauset et al. 2009). The post-processing, statistical tests, and the design of each plot presented in the results (frequency plot of each simulation, heatmap of SED difference location for each node of the tibia) were performed using MATLAB R2024a. The statistical significance level was set at *α* = 0.05.

## Results

### MIS area

The MIS values obtained after mesh morphing were as follows: 0.75 ± 0.26 mm2 for the QUAD muscle, 5.27 ± 0.33 mm2 for the TA muscle, 0.67 ± 0.26 mm2 for the SM muscle, 0.04 ± 0.01 mm2 for the EDL muscle, 0.48 ± 0.12 mm2 for the FDL muscle, 1.88 ± 0.06 mm2 for the ST muscle, and 3.74 ± 0.28 mm2 for the GA muscle.

### Effect of MIS area on SED for each muscle independently

**Fig. 3:**
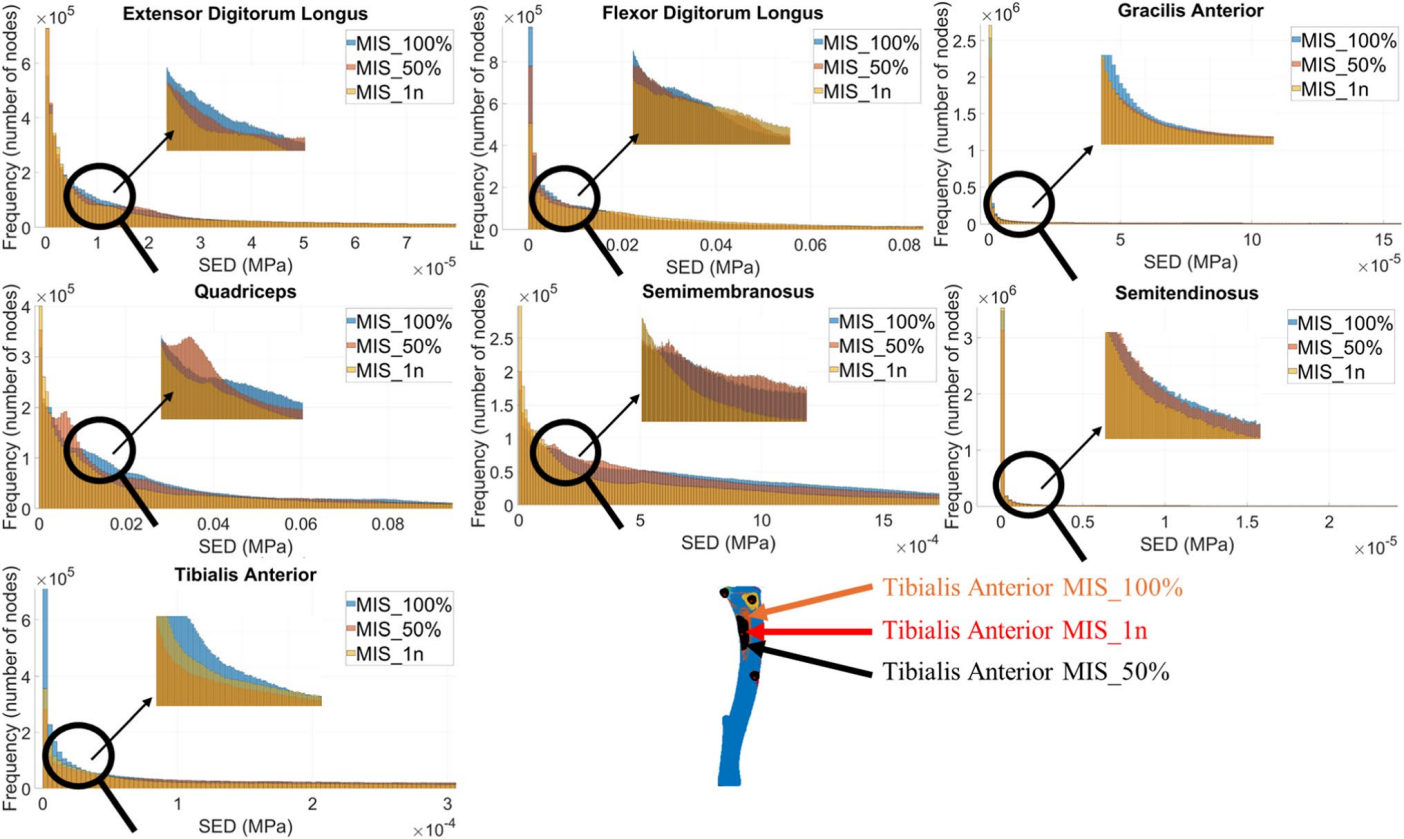
SED distribution for each muscle of one of the six tibiae simulated. This figure shows the Pareto distribution obtained for each configuration justifying the choice of analysing the median, mean and 95th percentile of the SED values. Subfigures highlight the differences between the distributions of the SED values calculated for the micro-FE with different MISs

**Fig. 4:**
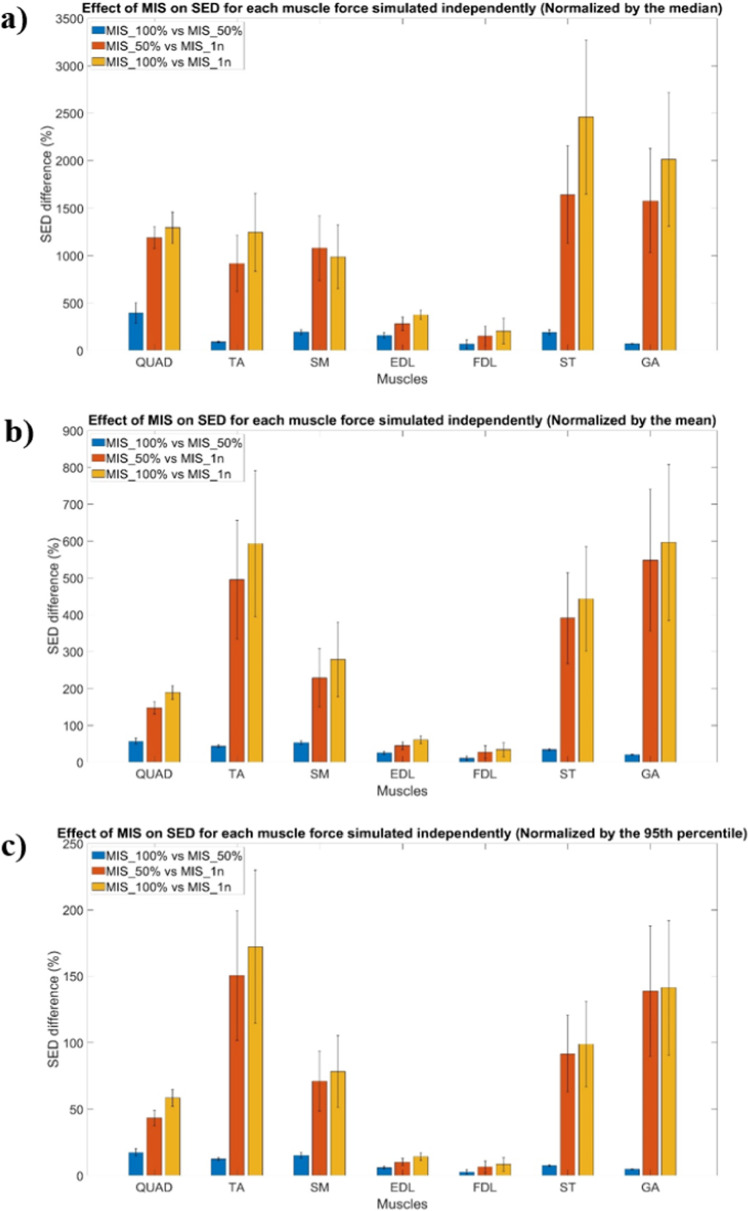
Normalised SED differences among the three different MIS conditions tested for each muscle independently. The normalised SED difference reported here corresponds to the difference calculated with Eq. 1. The differences obtained by the three normalisation methods are presented here, including (**a**) the normalisation by the median of the SED values in the reference condition, (**b**) the mean of the SED values, and (**c**) the 95th percentile of the SED values. The reference condition was the condition MIS_100%, except for the comparison between MIS_50% and MIS_1n, where MIS_50% was considered as the reference condition. The values presented here correspond to the mean values obtained for the six tibia micro-FE models, and the bar represents the associated standard deviation

**Fig. 5:**
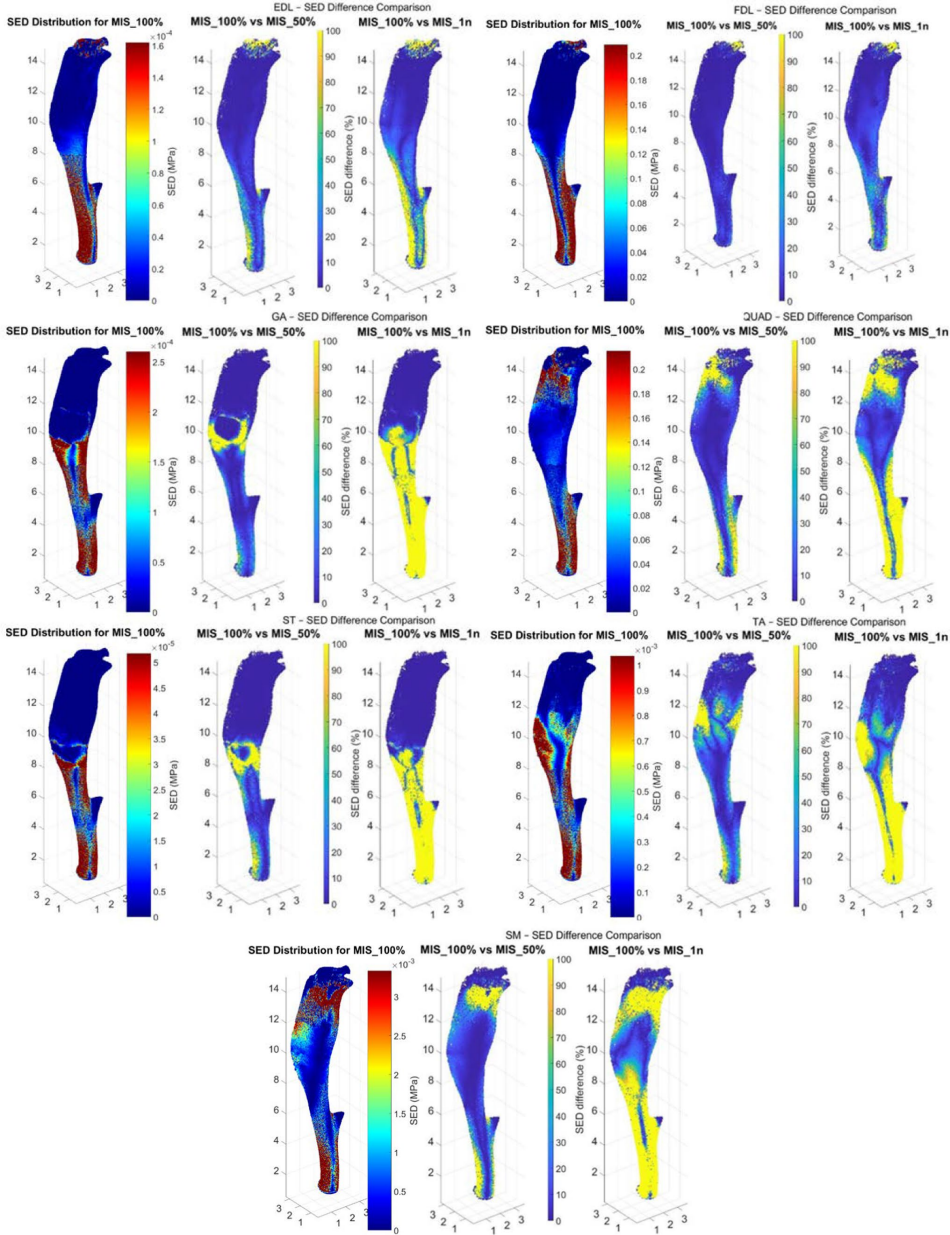
3D heatmap of the anatomical location of the largest SED differences for each muscle simulated independently. This figure shows the normalised SED difference obtained for each node of the tibia for one of the six micro-FE models used. The high SED differences visible in the proximal part of the tibia (in yellow) correspond to the anatomical location of the MIS of each muscle, respectively. These graphs show the normalisation by the mean, but the heatmaps remain very similar when using the other two normalisation techniques. In the same way, the heatmaps of the differences between the MIS_50% and MIS_1n conditions are not presented in this graph but are similar to the difference between MIS_100% and MIS_1n. The SED distribution for the condition MIS_100% is also visible on the left-hand side of each sub-plot

The Pareto distribution was confirmed for each simulation performed (P > 0.05) (Fig. 3). The percentage differences among the normalised SED values for the three MIS configurations are summarised in Table 1 and Fig. 4. Specifically, when normalised by the median, the SED differences ranged from 66.3 to 397.1% for the MIS_100% versus MIS_50% comparison, from 152.5 to 1,643.3% for MIS_50% versus MIS_1n, and from 205.3 to 2,459.0% for MIS_100% versus MIS_1n. When normalised by the mean, the differences varied from 10.8 to 56.8%, 27.7–548.4%, and 33.9–596.0%, respectively. Normalisation by the 95th percentile yielded differences ranging from 2.7 to 17.5% for MIS_100% versus MIS_50%, 6.8–150.5% for MIS_50% versus MIS_1n, and 8.4–172.1% for MIS_100% versus MIS_1n. In all comparisons, the SED differences were statistically significant (p < 0.0001). A heatmap showing the anatomical distribution of the largest SED differences within the tibia is presented in Fig. 5.

**Table 1:** Percentage SED differences among the different MIS cases, reported for each muscle (mean ± SD calculated for the six mouse tibiae). All differences were found to be significant (p < 0.0001)

	QUAD	TA	SM	EDL	FDL	ST	GA
	Differences normalised by the median (%)						
MIS_100% versus MIS_50%	397.1 ± 106.3	91.1 ± 6.2	193.3 ± 28.6	158.9 ± 29.8	66.3 ± 47.7	190.0 ± 25.8	70.2 ± 3.2
MIS_50% versus MIS_1n	1188.9 ± 115.5	917.2 ± 297.1	1078.5 ± 341.0	282.7 ± 70.7	152.5 ± 103.4	1643.3 ± 512.9	1578.3 ± 546.5
MIS_100% versus MIS_1n	1294.8 ± 165.6	1245.3 ± 411.1	985.7 ± 334.9	377.9 ± 48.3	205.3 ± 135.7	2459.0 ± 811.6	2012.7 ± 703.8
	Differences normalised by the mean (%)						
MIS_100% versus MIS_50%	56.8 ± 8.0	43.2 ± 3.4	53.3 ± 4.8	25.2 ± 3.7	10.8 ± 6.0	33.9 ± 2.4	20.5 ± 0.8
MIS_50% versus MIS_1n	147.7 ± 16.7	495.1 ± 161.1	228.8 ± 79.1	44.6 ± 10.3	27.7 ± 16.9	390.8 ± 123.0	548.4 ± 192.6
MIS_100% versus MIS_1n	189.6 ± 18.5	593.1 ± 198.2	278.3 ± 100.9	60.8 ± 10.0	33.9 ± 19.4	443.3 ± 142.3	596.0 ± 211.3
	Differences normalised by the 95th percentile (%)						
MIS_100% versus MIS_50%	17.5 ± 2.7	12.5 ± 1.2	15.2 ± 2.1	5.9 ± 0.9	2.7 ± 1.6	7.5 ± 0.6	4.8 ± 0.2
MIS_50% versus MIS_1n	43.2 ± 5.7	150.5 ± 48.9	71.2 ± 22.7	10.2 ± 2.6	6.8 ± 4.4	91.7 ± 28.9	138.7 ± 49.0
MIS_100% versus MIS_1n	58.4 ± 6.4	172.1 ± 57.7	78.2 ± 26.9	14.3 ± 2.6	8.4 ± 5.2	98.9 ± 32.0	± 50.5

### Effect of MIS area on SED for multiple muscles

The median absolute difference of SED (MAD_SED), normalised by the median SED, ranged from 96.9 to 217.2% across the stance phase for the MIS_100% versus MIS_50% comparison, from 864.0 to 308,470.0% for MIS_50% versus MIS_1n, and from 1135.2 to 489,760.0% for MIS_100% versus MIS_1n. When normalised by the mean SED, the MAD_SED ranged from 22.2 to 61.4%, 243.1–95,589.0%, and 323.6–122,050.0%, respectively. Normalisation by the 95th percentile yielded values ranging from 5.0 to 19.7%, 81.4–30,069.0%, and 102.4–34,199.0% for the same comparisons. The full set of percentage differences in SED for each normalisation method, comparison, and stance phase instant is presented in Table 2. For each comparison (i.e. SED distributions across the three MIS conditions at all time points and for all tibiae), statistical testing confirmed highly significant differences (p < 0.0001).

**Table 2:** Percentage SED differences among the different MIS cases (mean ± SD calculated for the six mouse tibiae) for the four instants of the stance phase (SP) simulated (5, 30, 55, and 95%). This table shows the results obtained for the modification of the MISs for the seven muscles simultaneously

	5% of SP	30% of SP	55% of SP	95% of SP
	Differences normalised by the median (%)			
MIS_100% versus MIS_50%	217.2 ± 33.1	179.8 ± 14.1	96.9 ± 6.0	140.6 ± 11.7
MIS_50% versus MIS_1n	864.0 ± 193.4	30 8470.0 ± 152 720.0	4780.7 ± 399.7	75 407.0 ± 35 880.0
MIS_100% versus MIS_1n	1135.2 ± 169.7	489 760.0 ± 243 310.0	7271.2 ± 580.1	121 050.0 ± 57 613.0
	Differences normalised by the mean (%)			
MIS_100% versus MIS_50%	61.4 ± 8.2	44.9 ± 4.2	22.2 ± 1.0	53.3 ± 5.4
MIS_50% versus MIS_1n	243.1 ± 53.0	93 589.0 ± 46 890.0	1476.0 ± 37.2	34 306.0 ± 16 260.0
MIS_100% versus MIS_1n	323.6 ± 64.0	122 050.0 ± 62 118.0	1664.3 ± 38.0	45 892.0 ± 21 746.0
	Differences normalised by the 95th percentile (%)			
MIS_100% versus MIS_50%	19.7 ± 3.0	12.6 ± 1.1	5.0 ± 0.3	13.3 ± 1.7
MIS_50% versus MIS_1n	81.4 ± 13.8	30 069.0 ± 14 559.0	361.0 ± 13.5	9496.1 ± 4509.7
MIS_100% versus MIS_1n	102.4 ± 12.0	34 199.0 ± 16 554.0	372.9 ± 14.2	11 539.0 ± 5476.1

## Discussion

The objective of this study was to determine the effect of muscle insertion surface (MIS) definition on local strain energy density (SED) values obtained from micro-FE models of the mouse tibia. To this end, micro-FE models of six mouse tibiae were developed from micro-CT images, and the MIS of the seven muscles contributing most to tibial loading were identified and applied in the simulations.

Significant differences in local SED values were observed across all comparisons (MIS_100%, MIS_50%, and MIS_1n) for each muscle, indicating a substantial effect of MIS size on SED distribution throughout the tibia. While such differences are somewhat expected, particularly for muscles with large insertion areas, this finding clearly demonstrates that applying muscle forces at single nodes (MIS_1n) is not appropriate when accurate estimation of local mechanical stimuli is required. This is especially relevant for bone adaptation models based on mechanoregulation.

The largest discrepancies in SED were observed near the MIS and in proximity to the fixed boundary conditions at the distal tibia (Fig. 5). This spatial pattern is consistent with the anatomical distribution of muscles, which generally span from the midshaft to the proximal region of the tibia. As a result, altering the MIS of proximal muscles significantly changes their moment arms, thereby impacting loading conditions, particularly in the distal tibia.

The impact of MIS variation on SED differed across muscles. For example, modifying the MIS of the quadriceps (QUAD) resulted in SED changes 6–8 times greater than those for the flexor digitorum longus (FDL), despite relatively similar MIS sizes and magnitude of the muscle forces. This surprising result may be explained by the anatomical locations of these muscles and the moments they apply to the tibia, which could amplify their effect on SED values. Nonetheless, percentage differences in SED were consistently large across all muscles and MIS comparisons. The smallest difference, 66.3%, normalised by the median, was observed for FDL when comparing MIS_100% to MIS_50%, and is still notably high. To the authors’ knowledge, only a previous study performed on the human femur (Polgar et al. 2003) compared the effect of using a realistic MIS_100% or nodal MIS_1n on the outputs of homogenised FE models. In that study small average SED differences between the two cases (~ 25%) were reported, compared to the much higher effects found in our study for the mouse tibia. This difference could be explained by considering the much different microstructure of the human femoral bone and the mouse tibia and the type of mesh used in the FE models (linear hexahedral elements in this study versus quadratic tetrahedral elements in that study). For example, the thin cortical bone in mice and the small element size used in this study could amplify the effect of the differences found between the models where the force is distributed on a large surface and the simplified model where the force is applied in one node or in a smaller MIS. These results underscore the need to model each muscle’s full MIS accurately when estimating local SED distributions, which are essential for informing bone adaptation models based on mechanical loading (Cheong et al. 2021; Levchuk et al. 2014; Pereira et al. 2015). The influence of MIS modification on multiple muscles remains non-negligible throughout the stance phase (Table 2). Even when percentage differences in SED values due to changes in multiple MIS definitions are comparable to those observed for individual muscles, differences exceeding 20% were found in all cases except when SED values were normalised to the 95th percentile. This suggests that inaccuracies in MIS definition may be less critical when mechanoregulation models are driven by high SED thresholds. Furthermore, the specific form of the mechanoregulation law implemented in computational bone adaptation models can mitigate the effects of large SED differences. For instance, a model that caps bone formation at a given SED threshold would naturally reduce the influence of extreme local SED values resulting from MIS simplifications. Notably, recent studies have shown that multiscale bone adaptation models can predict bone apposition in the mouse tibia with high accuracy when SED thresholds are set around the median SED values consistent with those reported in this study (Cheong et al. 2021). Therefore, in these cases, the use of realistic MISs is critical. Previous studies to predict bone adaptation in the mouse tibia using local mechanical stimuli calculated from micro-FE models have demonstrated that bone formation is strongly mechanoregulated, with up to 50–90% of bone apposition being related to SED (Cheong et al. 2020). However, these models are typically based on the mechanostat theory and implemented as threshold-driven mechanoregulation laws, where the biological response depends on whether the mechanical stimulus exceeds specific formation or resorption thresholds rather than on its magnitude. As such, the model is very sensitive to SED values around these thresholds. While previous studies have not explicitly quantified the effect of percentage variations in SED on predicted bone adaptation, the differences observed in the present study due to different muscle insertion surfaces (differences of 20–60% or higher) are likely to be sufficient to shift regions of bone tissue across these thresholds, thereby altering predicted spatial patterns of bone adaptation. It should be noted that the observed large percentage differences in SED can be due to the low SED values in regions of the tibia, leading to large relative differences that do not necessarily reflect substantial absolute SED changes. However, the trends observed in this study were consistent across different normalisation approaches, including median and 95th percentile scaling, suggesting that the reported differences are not solely driven by low baseline values. This is confirmed by the observation of significant SED differences also in regions exhibiting moderate to high SED values (Fig. 5), which are more relevant for bone mechanoregulation. Therefore, the overall conclusions regarding the sensitivity of SED distributions to MIS definition remain robust.

Some limitations should be considered for this study. First, the micro-FE models developed combined 3D models generated from micro-CT acquisitions performed on female mice with muscle forces estimated from gait analysis performed on male mice. This difference can affect the loading conditions implemented in the micro-FE models and the size and shape of the considered MIS. However, it is likely that the effect on the estimated SED values will remain similar. The lack of literature data on muscle and joint forces in female and male mice makes it difficult to understand the effect of this limitation on the final results. However, the data recently collected in our laboratory (unpublished data) on the three-dimensional kinematic and ground reaction force data on female C57BL/6 mice between 18 and 22 weeks of age, showed similar patterns and values as those collected in (Charles et al. 2018) on C57BL/6 6-week-old male mice. Future work will integrate information about muscle geometry, bone geometry, kinematics and ground reaction force on the same mice. Another limitation is the fact that only one speed of trotting gait was simulated in the MBD models (0.59 m.s^−1^, Charles et al 2018). Predicting muscle forces generated at different trotting speeds, which would likely include different joint kinematics, could result in different muscle forces and therefore different SED values. It should be noted that a linear micro-FE model with isotropic and homogeneous material properties was used for this study. This model has been previously validated against biomechanical experiments for the predictions of structural mechanical properties (Oliviero, Owen, et al., 2021a) and local deformation (Oliviero et al. 2018), showing that assumptions of material homogeneity at this dimensional scale are reasonable (Oliviero, Roberts, et al., 2021b). Moreover, when these models have been coupled to mechanoregulation laws, good predictions of bone apposition in the mouse tibia were found (Cheong et al. 2020). However, the effect of the MIS reported in this study could potentially differ if implemented with a more complex model including the fibula or using more complex constitutive laws (e.g. anisotropy, nonlinearities, poroelasticity).

Despite these limitations, this study is the first to quantify the effect of varying MIS sizes on SED values in micro-FE models of the mouse tibia. The findings clearly show that oversimplifying MIS implementation by using smaller insertion areas or point loads can lead to substantial errors in estimating local mechanical stimuli. Future research should focus on the importance of individualising MIS in finite element models and determining how accurately the surface must be defined to predict bone adaptation over time, particularly in the context of musculoskeletal diseases and related interventions.

## Data Availability

Example of the raw data and dataset used to generate the figures can be found in the following Figshare link (10.15131/shef.data.32324652). If the reader is interested in the raw data, they can contact the corresponding author.

## References

[CR1] Akay T, Tourtellotte WG, Arber S, Jessell TM (2014) Degradation of mouse locomotor pattern in the absence of proprioceptive sensory feedback. Proc Natl Acad Sci U S A 111(47):16877–16882. 10.1073/pnas.1419045111 25389309 10.1073/pnas.1419045111 PMC4250167

[CR2] Altai Z, Montefiori E, Van Veen BA, Paggiosi M, McCloskey EV, Viceconti M, Mazzà C, Li X (2021) Femoral neck strain prediction during level walking using a combined musculoskeletal and finite element model approach. PLoS ONE 16(2):e0245121. 10.1371/journal.pone.0245121 33524024 10.1371/journal.pone.0245121 PMC7850486

[CR3] Besl PJ, McKay ND (1992) A method for registration of 3-D shapes. IEEE Trans Pattern Anal Mach Intell 14(2):239–256. 10.1109/34.121791

[CR4] Bonewald LF (2011) The amazing osteocyte. J Bone Miner Res 26(2):229–238. 10.1002/jbmr.320 21254230 10.1002/jbmr.320 PMC3179345

[CR5] Bouxsein ML, Boyd SK, Christiansen BA, Guldberg RE, Jepsen KJ, Müller R (2010) Guidelines for assessment of bone microstructure in rodents using micro–computed tomography. J Bone Miner Res 25(7):1468–1486. 10.1002/jbmr.141 20533309 10.1002/jbmr.141

[CR6] Charles JP, Cappellari O, Spence AJ, Hutchinson JR, Wells DJ (2016) Musculoskeletal geometry, muscle architecture and functional specialisations of the mouse hindlimb. PLoS ONE 11(4):e0147669. 10.1371/journal.pone.0147669 27115354 10.1371/journal.pone.0147669 PMC4846001

[CR7] Charles JP, Cappellari O, Hutchinson JR (2018) A dynamic simulation of musculoskeletal function in the mouse hindlimb during trotting locomotion. Front Bioeng Biotechnol 6:61. 10.3389/fbioe.2018.00061 29868576 10.3389/fbioe.2018.00061 PMC5964171

[CR8] Charles JP, Kissane RWP, Askew GN (2024) The impacts of muscle-specific force-velocity properties on predictions of mouse muscle function during locomotion. Front Bioeng Biotechnol 12:1436004. 10.3389/fbioe.2024.1436004 39108597 10.3389/fbioe.2024.1436004 PMC11300213

[CR9] Cheong VS, Roberts BC, KadirkamanathanDall’Ara VE (2020) Bone remodelling in the mouse tibia is spatio-temporally modulated by oestrogen deficiency and external mechanical loading: a combined in vivo/in silico study. Acta Biomater 116:302–317. 10.1016/j.actbio.2020.09.011 32911105 10.1016/j.actbio.2020.09.011

[CR10] Cheong VS, Roberts BC, KadirkamanathanDall’Ara VE (2021) Positive interactions of mechanical loading and PTH treatments on spatio-temporal bone remodelling. Acta Biomater 136:291–305. 10.1016/j.actbio.2021.09.035 34563722 10.1016/j.actbio.2021.09.035

[CR11] Clauset A, Shalizi CR, Newman MEJ (2009) Power-law distributions in empirical data. SIAM Rev 51(4):661–703. 10.1137/070710111

[CR12] Elefteriou F, Yang X (2011) Genetic mouse models for bone studies—strengths and limitations. Bone 49(6):1242–1254 21907838 10.1016/j.bone.2011.08.021 PMC3331798

[CR13] Farage-O’Reilly SM, Cheong VS, Pickering E, Pivonka P, Bellantuono I, Kadirkamanathan V, Dall’Ara E (2024) The loading direction dramatically affects the mechanical properties of the mouse tibia. Front Bioeng Biotechnol 12:1335955. 10.3389/fbioe.2024.1335955 38380263 10.3389/fbioe.2024.1335955 PMC10877372

[CR14] Farage-O’Reilly SM, Cheong VS, Pivonka P, Kadirkamanathan V, Dall’Ara E (2025) Quantifying the local strain energy density distribution in the mouse tibia: the critical role of the loading direction. Biomech Model Mechanobiol. 10.1007/s10237-025-02011-z 10.1007/s10237-025-02011-z PMC12618303 40900216

[CR15] Frost HM (2003) Bone’s mechanostat: a 2003 update. Anat Rec A Discov Mol Cell Evol Biol 275A(2):1081–1101. 10.1002/ar.a.10119 10.1002/ar.a.10119 14613308

[CR16] Gkastaris K, Goulis DG, Potoupnis M, Anastasilakis AD, Kapetanos G (2020) Obesity, osteoporosis and bone metabolism. J Musculoskelet Neuronal Interact 20(3):372–381 32877973 PMC7493444

[CR17] Levchuk A, Zwahlen A, Weigt C, Lambers FM, Badilatti SD, Schulte FA, Kuhn G, Müller R (2014) The clinical biomechanics award 2012—presented by the European Society of Biomechanics: large scale simulations of trabecular bone adaptation to loading and treatment. Clin Biomech (Bristol, Avon) 29(4):355–362. 10.1016/j.clinbiomech.2013.12.019 10.1016/j.clinbiomech.2013.12.019 24467970

[CR18] Lorentzon M, Johansson H, Harvey NC, Liu E, Vandenput L, McCloskey EV, Kanis JA (2022) Osteoporosis and fractures in women: the burden of disease. Climacteric 25(1):4–10. 10.1080/13697137.2021.1951206 34319208 10.1080/13697137.2021.1951206

[CR19] Martin, R. B., Burr, D. B., & Sharkey, N. A. (1998). Skeletal tissue mechanics. Springer, New York. 10.1007/978-1-4757-2968-9

[CR20] Oliviero S, Giorgi M, Dall’Ara E (2018) Validation of finite element models of the mouse tibia using digital volume correlation. J Mech Behav Biomed Mater 86:172–184. 10.1016/j.jmbbm.2018.06.022 29986291 10.1016/j.jmbbm.2018.06.022

[CR21] Oliviero S, Owen R, Reilly GC, BellantuonoDall’Ara IE (2021a) Optimization of the failure criterion in micro-finite element models of the mouse tibia for the non-invasive prediction of its failure load in preclinical applications. J Mech Behav Biomed Mater 113:104190. 10.1016/j.jmbbm.2020.104190 33191174 10.1016/j.jmbbm.2020.104190

[CR22] Oliviero S, Roberts M, Owen R, Reilly GC, Bellantuono I, Dall’Ara E (2021b) Non-invasive prediction of the mouse tibia mechanical properties from microCT images: comparison between different finite element models. Biomech Modeling Mechanobiol 20(3):941–955. 10.1007/s10237-021-01422-y 10.1007/s10237-021-01422-y PMC8154847 33523337

[CR23] Pereira AF, Javaheri B, Pitsillides AA, Shefelbine SJ (2015) Predicting cortical bone adaptation to axial loading in the mouse tibia. J R Soc Interface 12(110):20150590. 10.1098/rsif.2015.0590 26311315 10.1098/rsif.2015.0590 PMC4614470

[CR24] Phillips ATM, Villette CC, Modenese L (2015) Femoral bone mesoscale structural architecture prediction using musculoskeletal and finite element modelling. Int Biomech 2(1):43–61. 10.1080/23335432.2015.1017609

[CR25] Polgar K, Gill HS, Viceconti M, Murray DW, O’Connor JJ (2003) Strain distribution within the human femur due to physiological and simplified loading: finite element analysis using the muscle standardized femur model. Proc Inst Mech Eng H 217(3):173–189. 10.1243/095441103765212677 12807158 10.1243/095441103765212677

[CR26] Rachner TD, Khosla S, Hofbauer LC (2011) Osteoporosis: now and the future. Lancet 377(9773):1276–1287. 10.1016/S0140-6736(10)62349-5 21450337 10.1016/S0140-6736(10)62349-5 PMC3555696

[CR27] Roberts BC, Arredondo Carrera HM, Zanjani-pour S, Boudiffa M, Wang N, Gartland A, Dall’Ara E (2020) PTH(1–34) treatment and/or mechanical loading have different osteogenic effects on the trabecular and cortical bone in the ovariectomized C57BL/6 mouse. Sci Rep 10(1):8889. 10.1038/s41598-020-65921-1 32483372 10.1038/s41598-020-65921-1 PMC7264307

[CR28] Salari N, Ghasemi H, Mohammadi L, Behzadi MH, Rabieenia E, Shohaimi S, Mohammadi M (2021) The global prevalence of osteoporosis in the world: a comprehensive systematic review and meta-analysis. J Orthop Surg Res 16(1):609. 10.1186/s13018-021-02772-0 34657598 10.1186/s13018-021-02772-0 PMC8522202

[CR29] Scheuren AC, Vallaster P, Kuhn GA, Paul GR, Malhotra A, Kameo Y, Müller R (2020) Mechano-regulation of trabecular bone adaptation is controlled by the local in vivo environment and logarithmically dependent on loading frequency. Front Bioeng Biotechnol 8:566346. 10.3389/fbioe.2020.566346 33154964 10.3389/fbioe.2020.566346 PMC7591723

[CR30] Seo J-W, Kang D-W, Kim J-Y, Yang S-T, Kim D-H, Choi J-S, Tack G-R (2014) Finite element analysis of the femur during stance phase of gait based on musculoskeletal model simulation. Bio-Med Mater Eng 24(6):2485–2493. 10.3233/BME-141062 10.3233/BME-141062 25226949

[CR31] Seth A, Hicks JL, Uchida TK, Habib A, Dembia CL, Dunne JJ, Ong CF, DeMers MS, Rajagopal A, Millard M, Hamner SR, Arnold EM, Yong JR, Lakshmikanth SK, Sherman MA, Ku JP, Delp SL (2018) OpenSim: simulating musculoskeletal dynamics and neuromuscular control to study human and animal movement. PLoS Comput Biol 14(7):e1006223. 10.1371/journal.pcbi.1006223 30048444 10.1371/journal.pcbi.1006223 PMC6061994

[CR32] Shane E, Burr D, Abrahamsen B, Adler RA, Brown TD, Cheung AM, Cosman F, Curtis JR, Dell R, Dempster DW, Ebeling PR, Einhorn TA, Genant HK, Geusens P, Klaushofer K, Lane JM, McKiernan F, McKinney R, Ng A, Nieves J, O’Keefe R, Papapoulos S, Howe TS, van der Meulen MCH, Weinstein RS, Whyte MP (2014) Atypical subtrochanteric and diaphyseal femoral fractures: second report of a task force of the American Society for Bone and Mineral Research. J Bone Miner Res 29(1):1–23. 10.1002/jbmr.1998 23712442 10.1002/jbmr.1998

[CR33] Shen Y, Huang X, Wu J, Lin X, Zhou X, Zhu Z, Pan X, Xu J, Qiao J, Zhang T, Ye L, Jiang H, Ren Y, Shan P-F (2022) The global burden of osteoporosis, low bone mass, and its related fracture in 204 countries and territories, 1990–2019. Front Endocrinol 13:882241. 10.3389/fendo.2022.882241 10.3389/fendo.2022.882241 PMC9165055 35669691

[CR34] Speirs AD, Heller MO, Duda GN, Taylor WR (2007) Physiologically based boundary conditions in finite element modelling. J Biomech 40(10):2318–2323. 10.1016/j.jbiomech.2006.10.038 17166504 10.1016/j.jbiomech.2006.10.038

[CR35] Valente G, Pitto L, Schileo E, Piroddi S, Leardini A, Manfrini M, Taddei F (2017) Relationship between bone adaptation and in-vivo mechanical stimulus in biological reconstructions after bone tumor: a biomechanical modeling analysis. Clin Biomech 42:99–107. 10.1016/j.clinbiomech.2017.01.017 10.1016/j.clinbiomech.2017.01.017 28131017

[CR36] Van Arkel RJ, Modenese L, Phillips ATM, Jeffers JRT (2013) Hip abduction can prevent posterior edge loading of hip replacements. J Orthop Res 31(8):1172–1179. 10.1002/jor.22364 23575923 10.1002/jor.22364 PMC3736148

[CR37] Van’T Hof, R. J., & Dall’Ara, E. (2025). Analysis of bone architecture in rodents using micro-computed tomography. In A. I. Idris (Ed.), Bone Research Protocols 2885, 519–547). Springer US. 10.1007/978-1-0716-4306-8_26 10.1007/978-1-0716-4306-8_26 40448778

[CR38] Yeam CT, Chia S, Tan HCC, Kwan YH, Fong W, Seng JJB (2018) A systematic review of factors affecting medication adherence among patients with osteoporosis. Osteoporos Int 29(12):2623–2637. 10.1007/s00198-018-4759-3 30417253 10.1007/s00198-018-4759-3

